# The value of inpatient music therapy for gynae-oncology palliative care in Singapore

**DOI:** 10.3389/fpsyt.2025.1577545

**Published:** 2025-05-27

**Authors:** Kayla C. Wong, Wendy L. Magee, Sylvia Mun, Yu Zhen Sim, Tanuja Nair, Komal Tewani

**Affiliations:** ^1^ Child Life, Art and Music Therapy Programmes, KK Women’s and Children’s Hospital, Singapore, Singapore; ^2^ Music Therapy Programme, Boyer College of Music and Dance, Temple University, Philadelphia, PA, United States; ^3^ Allied Health Office, KK Women’s and Children’s Hospital, Singapore, Singapore; ^4^ Palliative Medicine, Fellow, Academy of Medicine, Singapore (FAMS), KK Women’s and Children’s Hospital and Clinical Asst Prof, Duke-NUS, Singapore, Singapore

**Keywords:** music therapy, music interventions, palliative care, end of life, gynae-oncologic cancer

## Abstract

Gynae-oncologic cancer affects the female reproduction system. When cancer treatment can no longer offer a cure, palliative care becomes the focus. This study aimed to understand how music therapy is valuable for women undergoing inpatient gynae-oncologic palliative care in a Singapore hospital since current evidence demonstrated limited knowledge on supportive care for this population. Utilizing a modified grounded theory, participant interviews and therapist reflexive logs generated knowledge on how music therapy was valuable for this population. Women undergoing inpatient gynae-oncology palliative care in Singapore were recruited at a single acute hospital. Sixteen participants between the ages of 25 to 84 were purposively sampled. Emergent from data were two key phenomena representing participants’ perception of their music therapy session(s); (1) The Interpersonal and Intermusical Experiences in Music Therapy, (2) The Function of Music in Music Therapy. The presence of a music therapist who is culturally sensitive and reflexive in their practice was quintessential in connecting with and providing a non-judgemental space for the women to fully express appreciation of their lives, leading to the palliation of symptoms such as anxiety and low mood, while increasing focus and attention. This study offers insights into clinical care specifically for women through a multicultural lens. Music therapy is valuable in providing a comfortable space to address patients’ psychosocial and emotional needs such as providing a space to take a break from a difficult reality, celebrate life, and to process grief and other difficult feelings through verbal and musical means within just one to two sessions.

## Introduction

Cancer is one of the highest causes of death in middle and high income countries (1). The Centers for Disease Control and Prevention (CDC) defines Gynae-oncologic cancer as cancers of the female reproduction system (2). According to the Singapore Cancer Registry Annual Report (3), 13419 females passed away in Singapore from cancer between 2017 to 2021, of breast cancer (17.2%), cervical cancer (2.8%), uterus cancer (2.8%), and ovarian cancer (5.2%). When cancer treatment can no longer offer a cure, palliative care becomes the focus (1). Music therapy is defined as “the scientific use of music interventions within a therapeutic relationship” with a credentialed music therapist to address a range of functional and wellbeing needs (4). Through a holistic and biopsychosocial approach, music therapy can support palliative patients with coping and acceptance through musical means (5). A systematic review indicated that music interventions had the potential to reduce anxiety in patients with cancer (6). Music therapy has also been found to help with coping, emotional expression and regulation, acceptance, sleep quality, pain management, and distress through grief and bereavement (5, 7).

Singapore has expanded palliative care services with at least six inpatient hospices, many of which have employed music therapy services to support patients (8). However, little has been published about music therapy for palliative care in Singapore (9, 10). As such, its relevance for a Singaporean health culture warrants inquiry.

Expanding multicultural views within palliative care is important to ensure practice is relevant across cultural groups. Singapore is known as a multi-racial, multi-religious and multi-cultural society, and has an ethnic framework of Chinese-Malay-Indian-Others (CMIO) racial framework (11). Multiculturalism is often seen through the lens of Western logic and reasoning, which lean towards constitutions of “freedom, rights and democracy” without the consideration of “cultural otherness” which come from the involvement of transnationalism (12, as cited in 13). For example, a music therapy study in Taiwan detailed that the ethnic traditions and the conservative culture of the population impacted the way certain western models in palliative care could be applied (14). It is important that multicultural views within palliative care are expanded to ensure practice is relevant to more cultural groups. Postcolonial countries in Asia, such as India and Singapore, tend to embrace a diverse ethnic population with different languages, various cultural and religious practices, and different ways of practising life in society (12, 13) Such countries provide an expanded viewpoint for multiculturalism. As such, Singapore is aptly positioned to valuably expand multicultural viewpoints for palliative patients’ needs in the context of a music therapy practice.

Gender roles also play a part in how patients may view palliative care, as the socialization of gender roles may influence expectations at the end of life, impacting the support and communication that patients require of healthcare providers (15). For example, women are typically socialized to be caring and sensitive to others above themselves, contrasting with typically socialized men who may believe they have to be strong, stoic and resolved to fight until they reach a dead end (15). Women clearly have different care needs as compared to men (15) and current evidence shows that women with Gynae-oncological cancer are not receiving adequate supportive care (16, 17).

This study aimed to explore the value of music therapy in addressing gynae-oncology symptoms as part of palliative care within a value-based healthcare model. The value equation is condition-specific and multi-dimensional, understood as value equals quality over cost, with value being the improvement and experience of patients’ health outcomes, quality being the measured improvement in a person’s health outcomes, and cost being the amount that is required to achieve the improvement of patient’s health, including improved efficiency and demonstration of valued care (18–20).

### Research questions

What is the value of inpatient music therapy for Gynae-oncology palliative care as perceived by women in a Singapore acute setting.

Sub-question 1. Which Music Therapy experiences did participants find valuable?
Sub-question 2. In what ways were these experiences valuable for participants?

## Methods

This qualitative study explored the value of Music Therapy for gynae-oncology palliative care in a Singapore hospital. Consolidated Criteria for Reporting Qualitative Research was followed (21). The research protocol was approved by SingHealth Centralised Institutional Review Board (2021-2371). Participants were recruited from multidisciplinary referrals at an acute women’s hospital in Singapore (May 2021- April 2023) using purposive sampling (**Table 1**). At the point of recruitment by a Co-PI, each participant was given an information leaflet detailing the support that would be rendered by the Art and Music therapy services if they wanted to be part of the programme. If consent was gained, the Music Therapist would then provide a session for the participant.

**Table 1:** Inclusion and exclusion criteria for recruitment.

Inclusion Criteria	Exclusion Criteria
Must be under the care of the Women's Palliative Care Service (WPCS) and referred for Art and/or Music Therapy	Patients with severe hearing and visual impairments
Participants must be able to converse in English or Mandarin skills	Patients who are unable to converse in English or Mandarin
Participants must be able to have at least one interview after their Art and/or Music Therapy session (within 5 working days).	Patients who are cognitively impaired
Participants must be aged 21 years or older	Patients who are under the age of 21
Participants must have the ability to provide informed consent	Participants who are unable to provide informed consent

Two music therapists were involved in providing music therapy sessions separately. Individual sessions were held by the participant’s bedside lasting 15 to 90 minutes, with a single session possibly involving more than one music intervention based on the patient’s needs [17-20]. Some participants were in a single room, while some participants were in a shared ward. If participants were in a shared ward, curtains would be drawn and music was played at a minimal volume to maintain a form of privacy for participants. Treatment fidelity was ensured as both music therapists followed the same guidelines and music therapy interventions (i.e. song choice, active improvisation, song composition, etc.) for adults in palliative care as detailed in Allen and Clements-Cortés (22).

Receptive interventions included Song Choice, Music for Reminiscence, Music for Relaxation, Song (Lyric) Discussion, Guided Imagery, and Somatic Listening (22). Active interventions included Active, Emphatic, and referential improvisation, Vocal and Instrumental Re-creation, Song Composition, Song Stories, Musical Autobiographies/Musical life Reviews, and Musical collages (22). A description of music therapy methods are provided in **Table 2**. The choice of intervention was made by the participant or deemed most appropriate by the Music Therapist to meet the patient’s assessed needs (22).

**Table 2:** Description of music therapy interventions adapted from Allen & Clements-Cortés, 2013.

Categories	Sub-categories	Description of intervention
Receptive Methods	Receptive Music Therapy	
	Song Choice	Preferred songs of patient to be chosen and listened to
	Music for Reminiscence	The retrieval of memories from music
	Music for Relaxation	The use of music to achieve physical comfort
	Song (Lyric) Discussion	Exploration of difficult emotions through the use of song lyrics/themes
	Guided Imagery	Music and imagery for inner work
	Somatic Listening	The use of musical tones and vibrations to address physiological functioning
Active Methods	Improvisation Music Therapy	
	Active Improvisation	The patient is encouraged to make music to explore their emotions by playing instruments freely
	Emphatic Improvisation	The music therapist improvises music in the moment that matches and brings compassion to the patient’s physical and mental state
	Referential Improvisation	The music therapist provides a verbal prompt (i.e. a story, topic, symbol) for improvisation to be based on
	Re-creative Music Therapy	
	Vocal	Singing or vocalizing familiar songs? individually or as a group
	Instrumental	Freely playing or learning an instrument individually or as a group
	Compositional Music Therapy	
	Song Composition	Writing of lyrics or instrumental parts for a new song
	Song Stories	The telling of a client’s story through the writing of a new song
	Musical Autobiographies/Musical life Reviews	Compilation of songs that tells their life story
	Music Collages	Compilation of songs that represents who the person is

Equipment involved in sessions included a guitar, electric keyboard, and percussion instruments such as hand drums and maracas and egg shakers. If a recording device was needed to record a patient’s written song as part of legacy work, then the patient’s handphone was usually used. A short post-session semi-structured interview was then conducted one on one with the participant by the music therapist. These interviews were recorded with the participant’s consent. English recordings were transcribed by clinical research coordinators (FI, NFS) and Mandarin recordings were translated to English (JS) and transferred to Nvivo for analysis. Music Therapists also kept reflexive logs of sessions which were analysed in Excel.

**Figure 1:**
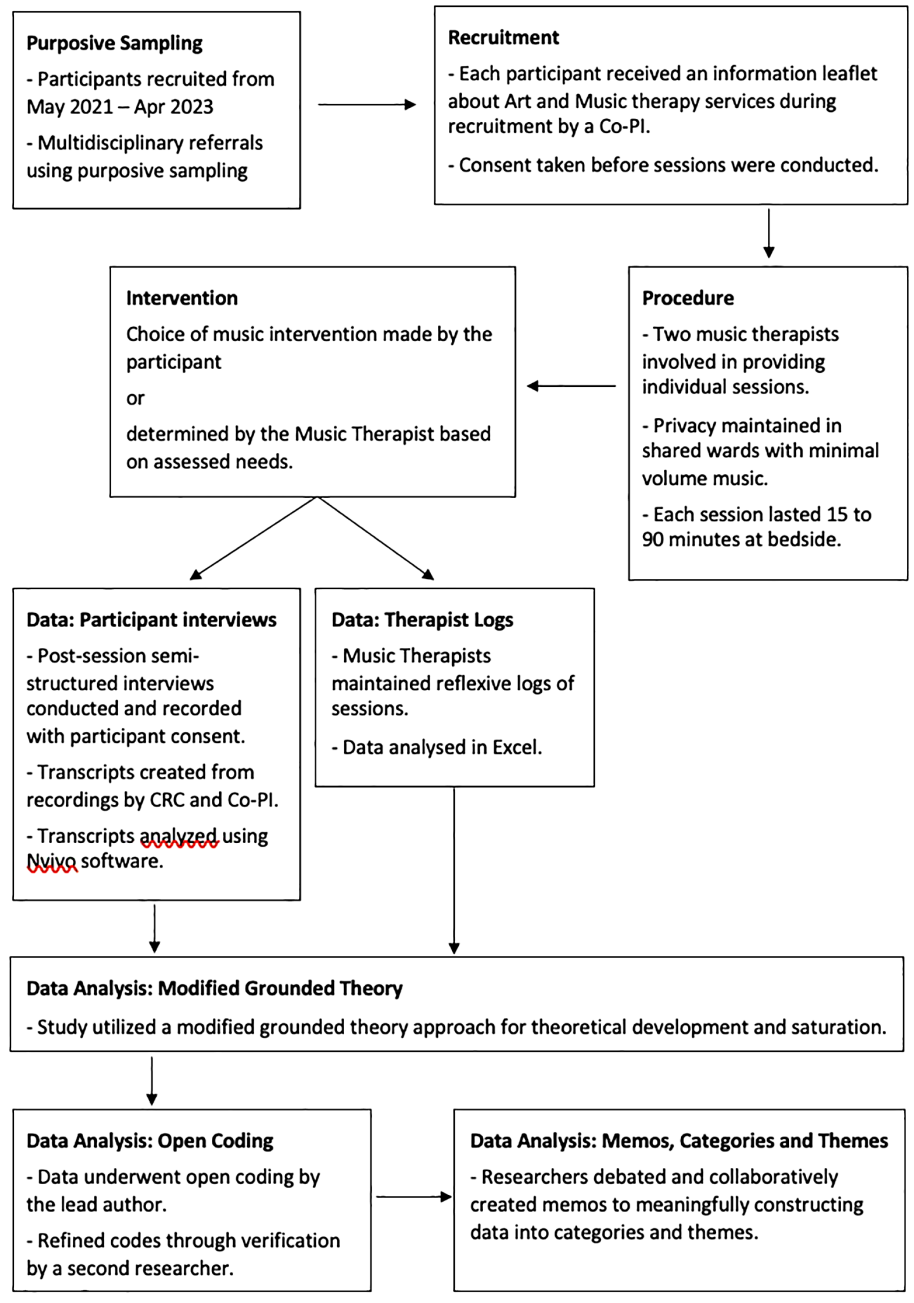
Qualitative study flow diagram illustrating the study design.

A modified grounded theory approach was used for this study. This means that grounded theory principles were followed up to the point of theory development (23). This approach allows for theoretical saturation to be attained, enabling theory construction to understand the value of inpatient music therapy based on the experiences of women receiving palliative care in a Singapore hospital (24). Data initially underwent open coding independently by the lead author (KW) who assigned codes and grouped data into preliminary categories based on participants’ words (25, 26). A second researcher (WM) then reviewed the preliminary coding before both researchers engaged in discussion on the given codes. Disagreements were discussed and both researchers jointly refined the codes, ensuring that data were assigned to a single code (25). The two researchers then debated the meaning of texts to understand the essence of the participants’ experiences until consensus was reached, creating memos as part of the process to construct meaning of the data into categories and themes (27) (**Figure 1**).

Trustworthiness was established through credibility, transferability and dependability and confirmability (28).

### Credibility

Three study members (KT, KW, JS) had involvement with the site and an understanding of the setting’s culture. Authenticity was achieved through data extracts that captured a real sense of participants’ voices. Groundedness of data was shown by the researchers creating memos based on higher level analyses of the data triangulated with the therapist reflexive logs.

### Transferability

Purposive sampling was used by only recruiting women undergoing inpatient gynae-oncology palliative care in Singapore to best understand the research question.

### Dependability

There was triangulation of multiple sources of data i.e. participant interviews, reflexive logs. Analysis was done by two study members (KW, WM).

### Confirmability

Data was linked to a range of participants.

## Results

22 participants were recruited for the study (**Table 3**). 16 participants received Music Therapy. 10 participants receiving a single session and six participants received two sessions. 12 participants were interviewed in English while four preferred Mandarin. 18 interviews and 20 reflexive log entries were used for analysis. Missing data were due to the participant being too unwell for an interview, or the therapist not completing their reflexive logs.

**Table 3:** Participant demographics.

Participant Demographics	Frequency
Age	
25-34	1
35-44	3
45-54	2
55-64	3
65-74	6
75-84	1
Race	
Chinese	10
Malay	2
Indian	1
Others	3
Marital Status	
Single	5
Married	10
Widowed	1
Number of Children	
No Children	5
1	6
2	4
5	1
Religion	
Buddhism	6
Christianity	4
Muslim	3
NA	2
Non Denomination	1
Diagnosis	
Uterine Cancer	6
Ovarian Cancer	5
Cervical Cancer	5

Within the single session, one or more music therapy interventions were used, based on participants’ choices or the music therapist’s recommendation. The most frequently used interventions were Song Choice, Music for Reminiscence, and Music for Relaxation under Receptive Music Therapy (see **Table 4**). Some interventions were not selected at all. Music preferences of participants included a range of genres of music, categorized by the first author as popular piano (e.g. Richard Clayderman), popular classics (e.g. Rainbow Connection), classical music, national songs, and hymns (e.g. Amazing Grace).

**Table 4:** Frequency of music therapy methods and interventions.

Music Therapy Interventions	Frequency
Receptive Music Therapy	42
Song Choice	19
Music for Reminiscence	10
Music for Relaxation	8
Song (Lyric) Discussion	5
Guided Imagery	0
Somatic Listening	0
Improvisation Music Therapy	4
Active Improvisation	2
Emphatic Improvisation	1
Referential Improvisation	1
Re-creative Music Therapy	3
Vocal	2
Instrumental	1
Compositional Music Therapy	3
Song Composition	2
Song Stories	1
Musical Autobiographies/Musical life Reviews	0
Music Collages	0

### Qualitative findings

Two major themes expanded into subthemes shed light on participants’ experiences of how the music therapy session gave value to their care while in hospital.

Major Theme 1: The Interpersonal and Intermusical Experiences in Music Therapy.

This major theme pertained to experiences within the active relationship between the participant, the music therapist, and the music within sessions. Feelings were evoked by the process that seemed particular to the music therapy session, as music is individualized by the music therapist for and with the patient, making this a highly personal experience. Within a musical experience, the opportunity for feelings and thoughts are discussed and explored between the music therapist and the participant regarding the context of their situation. The knowledge that one has limited time left elicits feelings of wanting to reflect and celebrate life and grieve the time they will lose. The music therapist is there to hold space for the participant as they are given the time to express themselves verbally and musically. Many of these experiences turned into opportunities for creating an artifact for participants’ loved ones to remember them by in the form of a song or musical legacy.

#### Subtheme 1.1: pre-existing attitudes towards music

Participants had existing relationships with music prior to engaging in music therapy, stemming from listening to the radio, listening to preferred music, or going to live music events with family and friends. Pre-existing relationships with music underpinned participants’ attitudes towards the music they chose to be played in the sessions. In these expressions of their attitude towards music, participants referred to feelings about the music, their perception of self, perception of what music could do for them, and the way they used music in their lives.

*I really interested in music. Any music just play out I know the songs already (P001)*.

*I like music so music to me, it’s just something that even when I’m at home, there’s always a video on, there must some music on, some sound. There must (be) a sound on, that’s all (P010)*.

#### Subtheme 1.2: feelings evoked by the music therapy process

Many participants detailed how they felt about the music created in the session and their perception of the changes elicited for them, such as calming them down, cheering them up, helping them to connect with their emotions, and feeling good. Through evoking memories of loved ones who were far away and providing an opportunity to talk about them, music elicited comfort and pleasure for the participants. Music therapy can also allow for grief to be processed within the music experience, through the emotions elicited by the music, and then allowing the patient to return to the state they prefer to maintain once the music experience comes to an end.

*Hmm … It calms me down … and make me connect with my own emotions better (P004)*.

*I feel very comfortable, very shiok (“Singlish” or Singapore-English for “pleasing” or “very enjoyable”) (P013)*.

*Those songs you sang, weren’t they really good songs? When it’s sung … I feel very … that … very calm ah…(P016)*.

#### Subtheme 1.3: perspective of music therapy experiences

Engaging in music therapy created a shift in participants’ perception of time. Many participants found the music therapy experience to be spontaneous and refreshing, yet touching and soothing, without feeling a sense of being rushed. These aspects of the music therapy experience can be particularly meaningful for a woman who is considering the end of her life. During music therapy, they found an escape, a sense of novelty, and felt a range of emotions. The sharing of a music experience between the participant and music therapist also promoted a sense of normalcy in life. Within the music, participants had the space to feel what they needed to feel and be nourished the way they needed to be.

*there is no rush … is comfortably pace (P004)*.

*sort of like err … get away from my current situation. I think it’s a good feeling ah. To have a break instead of err … thinking about why I’m facing this (P004)*.

*It’s a good thing la I get to join this because I said, at first I was like ahh, don’t want la. But then when I think back, eh why not I give it a try (P005)*.

#### Subtheme 1.4: music therapist’s presence

The participants expressed how the Music Therapist’s Presence impacted them in session. Sharing a musical experience was helpful for building rapport, allowing for a space where feelings could be expressed and support to be rendered within a therapeutic relationship. Many participants commented how the music therapist engaging the participant in live music by their bedside made the experience impactful. Participants also frequently commented on the music therapist’s gentle, soft voice. This was found in the data of participants who had experienced different music therapists, reflecting the strategy used by music therapists to adapt their voice to achieve a “therapeutic voice” to match the clients’ situation.

*Honestly say … your voice quite gentle you know. At least listen ah, you feel better (P007)*.

*You sing very well, I feel happy when you sing, that is, my mood is great, I feel very, what’s that, very stirred emotionally ah- (P016)*.

*Like just now you sing for me like eh! Touch my heart, I say wow it’s sooo relaxed (P019)*.

#### Subtheme 1.5: Legacy

This subtheme details the process of creating music for participants to leave a song for their loved ones to remember them by. Creating artifacts in the form of songs for family members gave participants an opportunity to communicate their feelings and wishes for their loved ones. Some of these songs were sung live or recorded for participants’ loved ones. This created a sense of hope that their loved ones would have something to remember them by, which in turn allowed participants to feel more relaxed.

*it’s relaxing for me to … err … play the music and compose the music for my family (P006)*.

*Today’s therapy, I can express something to my family people, no doubt I say that I’m blank, at least I can able to give me some thinking to do, to my family people, what I wish them (P010)*.

### Major theme 2: the function of music in music therapy

Music in music therapy functions by supporting patients from a holistic perspective. For example, by focusing deep attention on oneself, eliciting memories from the past, expressing who they are as a person, and understanding what they have contributed to their community. Music was an avenue for participants to create by playing instruments and recreate songs they enjoyed with the music therapist supporting them via live music.

#### Subtheme 2.1: embodiment of music

This subtheme describes how the process of experiencing music in a therapeutically targeted music intervention works. Listening to music in music therapy allowed for participants to become fully attentive. Through this process, they became introspective, entering into a state of personal reflection, expressing the parts of their inner world that they enjoy. This process of heightened self-awareness refocuses their attention on their emotional state rather than their physical state. Music is a cathartic experience, facilitating relaxation and the release of difficult feelings, along with the enjoyment of music. Music can be used as a container, allowing the patient to feel what they need to feel within the music experience. The patient can return to their preferred state of composure outside of the music experience. The creation of music in dialogue with the music therapist elicited novel experiences, perhaps allowing for the person to return to a child-like state which can help them to become more vulnerable to enable processing difficult emotions.

*all the sadness in … in in me … like everything is. like released (P005)*.

*because of the music therapy … I can express how, how is, how’s my feeling (P006)*.

*It makes me less anxious and uhh, basically the thoughts is, umm, I tend to overthink. But the overthinking, the focus is changed, the focus on the beautiful music (P016)*.

#### Subtheme 2.2: positive reminiscence

Positive Reminiscence was evoked by engaging in music. In particular, the use of patient-selected songs elicited long-term memories from across the lifespan, starting from childhood. Music facilitated reflections on their family, culture and life, including many meaningful experiences. Music-related experiences are often tied to memories involving an emotional connection with one’s background and others, be it family or friends, allowing for memories to be recalled, shared and reflected upon.

*Yes, those benefits. Those songs are very meaningful la, very meaningful. Reminds me of family la, happiness ah, like that la (P016)*.

*This musical session brought back a lot of memories especially… (silence) especially in music lah especially in music brought back a lot of memories that I will never forget. Childhood days’ ah (P018)*.

*these songs of ours are from really long ago, really old, in Singapore, there are so many people who love to sing these songs. These songs are more, are more well-liked by everyone la (P016)*.

#### Subtheme 2.3: clinical songwriting

Participants described how the making of music within the creative process in Clinical Songwriting was meaningful for them. It allowed them to feel relaxed, have their voices heard, have a sense of autonomy through their self-expression, and have a way to leave a “piece” of themselves for their loved ones. Songs created in the process of Clinical Songwriting provided a product that could form a musical legacy.

*I liked expressing, I liked the part where I express my words, the way we composed the song, yea and umm we managed to give it a melody. <laugh> and make it into a song even if just a short, a few, a few sentences that is very meaningful and memorable (P008)*.

*It’s just by simple words, and then can just make people understand what you want. Just simple lines and just what you want. Just simple song that’s all (P010)*.

Results from major themes and subthemes were superimposed into the value-based healthcare model to develop overarching themes. Under value, music therapy was found to support patients with adequate space and containment to escape worries, celebrate life, process grief and other difficult feelings, and express themselves verbally and musically. For quality, patient reported outcomes noted music therapy to be a meaningful experience, helping to improve mood and relaxation, comfort and pleasure, reduce anxiety, increase focus and attention, and aid in the releasing of difficult emotions. Finally, the influence of cost was demonstrated in how shared musical experiences helped expedite the building of rapport, allowing for positive outcomes to be achieved within lesser sessions.

## Discussion

### Music therapy as value-based healthcare

Based on the participants experiences in music therapy, we can see that the value of music therapy included having the option to offer different levels of therapy based on how the patient chose to engage in music therapy, be it to escape reality or to process their grief. Bruscia (29) lists four levels in medical music therapy; auxiliary, augmentative, intensive, and primary. In palliative care, two levels are described as relevant for application, augmentative and intensive (29, 30). In the augmentative level, the music therapist can help to reduce anxiety and provide a distraction before or after procedures through music experiences (29, 30). In the intensive level, music experiences are facilitated by the music therapist to help the patient process matters that arise surrounding their end of life (29, 30). Based on the patient’s readiness, the music therapist was able to support patients with adequate space and containment to escape worries, celebrate life, process grief and other difficult feelings, and express themselves verbally and musically.

A quality of music therapy can consider participant reported outcomes such as music therapy being a meaningful experience, helping to improve mood and relaxation, comfort and pleasure, reduce anxiety, increase focus and attention, and aid in the releasing of difficult emotions. Another quality that participants valued was the creation of musical artifacts to leave for their loved ones. They wondered at the speed of the process and how it was meaningful for them, while not feeling rushed. With most patients having been able to benefit greatly within only one or two sessions, music therapy optimizes the outcomes that can be achieved with a minimal number of clinical contacts.

Music Therapy is an inexpensive intervention that uses a patient-centred approach to care for patients (7). While this programme was funded by grants, a sustainable view in cost can consider how a shared musical experience may help expedite the building of rapport between patient and therapist. A good rapport within the therapeutic relationship is quintessential to having positive outcomes in therapy (31). Most patients were able to benefit greatly within only one or two sessions. Thus, music therapy optimizes the outcomes that can be achieved with a minimal number of clinical contacts. The Singapore Ministry of Health announced funding palliative care patients in hospital, home settings, and hospice day care settings (32). This research supports the inclusion of music therapy as part of standard palliative care so that patients can tap into resources such as government subsidies that might optimize their palliative care experience. While a limitation of this study was that it did not collect any cost savings data, this research is a first step towards gaining a deeper understanding of the value of music therapy as defined by the recipients of music therapy themselves. This can help shape future research measuring value using the value-based healthcare model.

### Women’s needs

Participants shared that music therapy gave them a voice and especially valued the ability to create a piece of music for their family to remember her by, which in turn helped them feel less anxious, more relaxed. In most societies, women take on a caregiving role, providing a lifetime of informal care with minimal recognition for their efforts (15). It has also been reported that women have commonly been undertreated for their symptoms due to the societal belief that women tend to exaggerate their pain due to anxiety and emotional distress (15). Through its holistic approach to care, music therapy provides women with a space to fully express themselves with no judgement, and for the women to have a space to feel valued for who they are and what they have done in their lives. In addition, Research has indicated that the sensitive and sentimental nature of being a woman may lead them to respond more readily to music (33).

### Multiculturalism

Participants were especially grateful that through the music therapy process, they were able to express and release their emotions, enabling them to reflect on their place in their community and contribute something to their family. This is reflective of a collective culture, where being part of a wider group is highly valued (34), and a sense of camaraderie where one can contribute and experience reciprocity with their community brings about feelings of pleasure and comfort (35). While music can elicit the expression of emotions, it can also help to contain intense emotions (36). This is helpful for working with women of Asian descent as being in control of emotions in order to preserve harmonious relationships within the community is an important Asian value to be considered (37).

Furthermore, women are usually socialized to not want to burden others (15), and so naturally would want to be in control of perceived difficult emotions. The definition of well-being is subjective based on the cultural identity and values of the person (38). As such, music therapists working with women in palliative care must provide individualized care and practice with cultural sensitivity and reflexivity to provide optimal care. This study only included patients who could communicate in English and Mandarin, proving to be a limitation in collecting rich multicultural data. Future studies can consider being intentional about collecting data from a wider range of mother tongues subject to resources available.

### Preferred music therapy interventions

This study found that the most frequently used interventions were Song Choice, Music for Reminiscence, and Music for Relaxation, allowing for comfort and pleasure, the releasing of difficult emotions, and the creation of musical artifacts to leave for their loved ones. These led to outcomes such as improved mood and relaxation, reduction of anxiety, and increased focus and attention.

#### Song choice

Horne-Thompson et al. (39) found that in 45% of Music Therapy sessions, patients preferred song sharing as the most prominent Music Therapy intervention. Aligned with those findings, Gallagher et al. (40) found that, live, patient-preferred music had the best effects for patients. Similarly, the findings of this study suggest that women in Singapore prefer Song Choice as it was the most popular music intervention chosen. Song Choice is a likely natural tool for building rapport, and so almost every participant would have experienced a music therapist offering them a Song Choice, with the question that is most commonly asked in a session “do you have a song that is meaningful to you?” Song Choice involves the music therapist encouraging the patient to choose and listen to their preferred songs (22). This is important in palliative care work as the use of this intervention can lead to the realization of many other goals such as reminiscence, emotional expression, and relaxation (22). This is reflective of the participants experience, expressing that the music made them feel like their feelings were released and that they have a greater sense of relaxation. The music therapist must ensure that Song Choice is introduced appropriately to avoid any harm to the patients, such as the danger of eliciting negative emotions in patients who may be mentally unaware or unstable (22).

#### Music for reminiscence

Music for Reminiscence relates to the music therapist prompting the patient to retrieve memories and recount them in a therapy setting (22). Music for Reminiscence can help to facilitate conversation between the patient and the therapist or their family, allowing for meaningful memories to be discussed, which in turn can help to improve mood and alleviate pain (22). Participants reflected on how meaningful the songs were, bringing back memories and reminding them of family. Music for Reminiscence can also help with the creation of a legacy piece that patients can leave for family, for example, a musical life review highlighting outstanding aspects of the patients life journey (22).

#### Music for relaxation

Music for Relaxation involves the use of music to optimize physical comfort (22). Music played for this goal should be predictable and steady in tempo and dynamics (22). Studies have shown that music interventions can lead to reductions in heart rate, respiratory rate and blood pressure (6). These Receptive Music Therapy methods are also most suitable for this population as many of patients are seeking comfort at this stage and receptive methods do not require them to be physically active to participate actively. This is aligned with participants reflections on how comfortable and pleasurable the music made them feel when the music therapist sang to them. Music can be useful for different levels of therapy, be it for escaping worries or processing difficult feelings. Music therapy should be tailored to what the patient is ready for and not for any other agenda i.e. therapist’s agenda to process deep and difficult emotions.

### Music therapist’s presence

The music therapist’s presence had impact for participants in the session, with particular emphasis on the music therapist engaging the participant in live music by their bedside. Participants reflected upon the two music therapist’s tone and timbre of voice. This is the therapeutic voice to resonate with the clients’ needs i.e. people who are dying or pondering death. The therapeutic voice is different from the voice of a vocal performer. The music therapist is sensitive to modifying vocal projection for the environment and situation of the patient in hospital based on their physical and emotional status, facilitating connection and validation to the patient, allowing them to feel deeply. Music therapists are trained to attune and emotionally resonate with the patient through music to provide patients an opportunity for meaningful moments where they can feel heard and explore inner feelings (41).

### Strength and limitations

This study is the first in Singapore to discuss the value of inpatient music therapy for women in palliative care. This study’s impact on practice includes recommendations for music therapists working with this population to adopt a non-judgmental, culturally sensitive and reflexive practice, enabling the ability to hold space for patients. This would allow patients a sense of normalcy in their situation, leading to them to express themselves adequately and benefit from physiological and emotional outcomes. Limitations of this study include having a small sample size, limiting the ability to compare subgroups that could provide further understanding on the impact of multiculturalism, age group, religion, marital and parental status. Future studies can aim to deepen insights on how these variables impact outcomes within the context of music therapy. Prospective studies can also focus on providing measurable evidence on the effectiveness of music therapy.

## Conclusion

The holistic nature of music therapy demonstrated the ability to effectively increase focus and attention and reduce symptoms like anxiety and low mood by providing a space to take a break from a difficult reality, celebrate life, and process grief and other difficult feelings through verbal and musical means. These outcomes were able to be achieved with just one or two clinical contacts. The presence of a music therapist who is culturally sensitive and reflexive in their practice was quintessential in connecting with and providing a non-judgmental space for the women to fully express themselves and take time to appreciate their lives.

## Data Availability

The raw data supporting the conclusions of this article will be made available by the authors, without undue reservation.
